# Effects of GABA and Vigabatrin on the Germination of Chinese Chestnut Recalcitrant Seeds and Its Implications for Seed Dormancy and Storage

**DOI:** 10.3390/plants9040449

**Published:** 2020-04-03

**Authors:** Changjian Du, Wei Chen, Yanyan Wu, Guangpeng Wang, Jiabing Zhao, Jiacheng Sun, Jing Ji, Donghui Yan, Zeping Jiang, Shengqing Shi

**Affiliations:** 1 State Key Laboratory of Tree Genetics and Breeding, Key Laboratory of Tree Breeding and Cultivation of State Forestry and Grassland Administration, Research Institute of Forestry, the Chinese Academy of Forestry, 1958 Box, Beijing 100091, China; duchnagjian@126.com (C.D.); 15764356257@163.com (W.C.); 15732153437@163.com (Y.W.); sjc9867@163.com (J.S.); jijing0401@163.com (J.J.); 2 Institute for Pomology, Hebei Academy of Agriculture and Forestry Sciences, Changli 066600, China; wangguangpeng430@163.com; 3 College of Forestry, Hebei Agricultural University, Baoding 071001, China; zhaojiabing0310@163.com; 4 Key Laboratory of Forest Ecology and Environment of National Forestry and Grassland Administration, Research Institute of Forest Ecology, Environment and Protection, the Chinese Academy of Forestry, 1958 Box, Beijing 100091, China; yandh@caf.ac.cn (D.Y.); jiangzp@caf.ac.cn (Z.J.)

**Keywords:** chestnut, GABA, seed germination, carbon metabolism, nitrogen metabolism

## Abstract

Recalcitrant chestnut seeds are rich in γ-aminobutyric acid (GABA), which negatively regulates adventitious root development by altering carbon/nitrogen metabolism. However, little is known regarding the role of this metabolite in chestnut seeds. In this study, we investigated the effects of GABA changes on the germination of chestnut seeds treated with exogenous GABA and vigabatrin (VGB, which inhibits GABA degradation). Both treatments significantly inhibited seed germination and primary root growth and resulted in the considerable accumulation of H_2_O_2_, but the endogenous GABA content decreased before germination at 48 h. Soluble sugar levels increased before germination, but subsequently decreased, whereas starch contents were relatively unchanged. Changes to organic acids were observed at 120 h after sowing, including a decrease and increase in citrate and malate levels, respectively. Similarly, soluble protein contents increased at 120 h, but the abundance of most free amino acids decreased at 48 h. Moreover, the total amino acid levels increased only in response to VGB at 0 h. Accordingly, GABA and VGB altered the balance of carbon and nitrogen metabolism, thereby inhibiting chestnut seed germination. These results suggested that changes to GABA levels in chestnut seeds might prevent seed germination. The study data may also help clarify the dormancy and storage of chestnut seeds, as well as other recalcitrant seeds.

## 1. Introduction

Chestnut (genus *Castanea*; family Fagaceae), which is a major nut crop in East Asia and Southern Europe, is unique among temperate nut crops because its seed is starchy rather than oily, making it ecologically and economically valuable [1,2]. The production of American chestnut, however, has been severely affected in North America by chestnut blight due to *Cryphonectria parasitica* [3,4]. As a perennial crop, chestnut is not only used as an important starch-based food product consumed by people living in rural areas [5,6], but also as a potential functional food because it is a rich source of bioactive compounds, including phenolics [5]. Additionally, chestnut seeds contain a considerable abundance of γ-aminobutyric acid (GABA) [7], which is the key component of the GABA shunt crucial for carbon and nitrogen metabolism in plants [8]. Previous studies have also identified GABA-enriched functional foods [9,10]. However, there is still relatively little information regarding GABA functions affecting chestnut seed activities, including germination.

Seeds, including orthodox and recalcitrant seeds [11], play a major role in agriculture, serving as food and feed, as well as plant propagation units. Seed germination is influenced by internal metabolic changes [12,13] and a complex process in which starch-degrading α-amylase and various proteases are activated to decrease the total dry matter content [14]. During germination, starch and proteins are degraded into smaller molecules, such as soluble sugars and free amino acids [15], resulting in a significant increase in the free amino acid content [16]. Previous studies proved that GABA levels increase during the germination of barnyard millet [16] and wheat [17,18] seeds under normal conditions, as well as wheat seeds under saline conditions [18]. The accumulation of endogenous GABA in dry seeds facilitates early metabolic reorganization during germination [19]. Moreover, exogenous GABA reportedly affects the seed germination process in barley [20] and *Haloxylon ammodendron* [21] under normal conditions. It also modulates respiration during the germination of *H. ammodendron* seeds [21] and regulates H_2_O_2_ production in *Caragana intermedia* [22] and poplar [23] in response to salt stress. The application of exogenous GABA can also mitigate salt-mediated damages by enhancing starch catabolism and the use of sugars and amino acids [24] or by enhancing the antioxidant system to induce the accumulation of phenolic compounds during seed germination [25]. Additionally, an exogenous GABA treatment can delay the loss of titratable acidity and malate, thereby maintaining the quality of stored apple fruits [26]. These studies confirmed that GABA can affect seed germination or fruit storage by altering the metabolism of carbon and nitrogen, as well as reactive oxygen species (ROS).

Chestnut seeds are recalcitrant and exhibit dormancy [27,28], which differentiates them from most other recalcitrant seeds [29]. This dormancy increases the shelf-life of the seeds used as food and enables the seeds to survive unfavorable winter conditions so they can germinate in the following spring. The normal germination of chestnuts is vital for their use as rootstock seedlings during grafting [30]. Earlier reports indicated that the germination rates of 56 selected types and/or cultivars of European chestnut seeds were 17.6–86.6% [31] and 51.6–97.3% [32] in two regions in Turkey, depending on their dormancy-breaking time due to stratification temperatures [28,31,32]. Similar to orthodox seeds, the dormancy of recalcitrant seeds appears to be induced by abscisic acid (ABA) [29,33], which is abundant in chestnut seed coats [34]. In contrast, the contents of the ABA antagonist GA_3_ are high in embryos and cotyledons [34]. Thus, different exogenous treatments have been used to increase germination rates, including an H_2_O_2_ treatment [35] and the application of GA_3_ [30,34]. Notably, an earlier investigation demonstrated that GABA is a major amino component associated with the high accumulation of several amino acids during chestnut seed germination [36], suggesting there is a close relationship between GABA and the recalcitrant chestnut seeds.

The effects of GABA on chestnut seed germination remain unclear. Thus, on the basis of our previous findings that GABA regulates stress responses [22,23] and adventitious root development [37], in this study, we treated Chinese chestnut (*Castanea mollissima*) cultivar “Yanshanzaofeng” seeds with GABA and vigabatrin (VGB; a specific GABA transaminase inhibitor) to investigate seed germination changes, as well as the central carbon/nitrogen metabolic activities, which demonstrated that both treatments inhibited chestnut seed germination and might by altering the balance of carbon and nitrogen metabolism, which would provide a better understanding for elucidating the role of GABA during the storage and germination of recalcitrant chestnut seeds. 

## 2. Materials and Methods

### 2.1. Plant Materials and Treatments

Chinese chestnut (*Castanea mollissima*) cultivar “Yanshanzaofeng” seeds were collected from healthy trees growing in the core chestnut-producing region of Qianxi county, Tangshan city, Hebei province, China, after which they were stored at 0–1 °C until further use. Relatively uniform chestnut seeds were washed five times with sterile water and air-dried. The seeds were then soaked in sterile water (control/CK), 10 mM GABA (lab use only; Sigma-Aldrich, St. Louis, MO, USA), or 100 μM vigabatrin (VGB; lab use only; MedChem Express, Monmouth Junction, NJ, USA) for 15 h at 25 °C before they were placed evenly on a tray (33.5 cm × 26 cm × 11 cm) containing sterilized sand. A germination test was conducted in a climate chamber with a 16 h light (25 °C): 8 h dark (20 °C) cycle and 60% relative humidity. The seed germination rate was calculated, and the root length was measured after 2, 5, 8, 15, and 30 days. Five seeds per replicate were collected at 15 h before the treatment (t0; i.e., the time-point when the seed imbibition was initiated) and at 0, 48, and 120 h after sowing. After discarding the seed coats, the kernels were ground into a powder and stored at −80 °C for the subsequent physiological measurements. Each treatment was completed with three replicates, each comprising 50 seeds.

### 2.2. Calculation of the Seed Germination Rate

Seed radicle protrusion was used as the criterion for judging germination. Germinated seeds were counted at the designated treatment times, after which the germination rate was calculated based on the ratio of the number of germinated seeds and the total number of seeds in each treatment.

### 2.3. Measurement of Reactive Oxygen Species

The H_2_O_2_ content was measured as previously described [38]. Briefly, 0.1 g fresh powder were dissolved in 1 mL acetone and then thoroughly mixed on ice. After a centrifugation (8000× *g* for 10 min at 4 °C), the supernatant was mixed with a titanium sulfate solution and concentrated ammonia. After another centrifugation (4000× *g* for 10 min at 25 °C), the sediment was dissolved in concentrated sulfuric acid and incubated at room temperature for 5 min. The absorbance of the reaction solution was measured at 415 nm, and the H_2_O_2_ content was recorded as μmol/g fresh weight (FW).

### 2.4. Measurement of Soluble Sugars and Starch

The total soluble sugar content was measured with a commercial assay kit (Comin Biotechnology, Suzhou, China) based on the anthrone-sulfuric acid method as previously described [39]. Briefly, 0.1 g fresh powder were dissolved in 1 mL sterile water and heated for 10 min at 95 °C. After a centrifugation (8000× *g* for 10 min at 25 °C), the diluted supernatant was added to a reaction mixture comprising anthranone in ethyl acetate and a concentrated sulfuric acid solution and then heated for 10 min at 95 °C. The absorbance of the reaction solution was measured at 620 nm, and the soluble sugar content was recorded as % FW.

The starch content was quantified with a commercial assay kit (Comin Biotechnology) based on a previously described method [40]. Briefly, 0.1 g fresh powder were dissolved in 1 mL ethanol solution and heated at 80 °C for 30 min. The solution was centrifuged (3000× *g* for 5 min at 25 °C), and the sediment was dissolved in 0.5 mL sterile water and heated at 95 °C for 15 min. After cooling, 0.35 mL perchloric acid and 0.85 mL sterile water were added, and the resulting solution was thoroughly mixed and then centrifuged (3000× *g* for 10 min at 25 °C). A 50 μL aliquot of the solution was mixed with 250 μL reaction mixture (3.75 mL anthranone solution and 21.25 mL concentrated sulfuric acid) and then heated at 80 °C for 10 min. The absorbance of the reaction solution was measured at 620 nm, and the starch content was recorded as % FW.

### 2.5. Measurement of Soluble Proteins and Total Amino Acids

The total soluble protein content was measured with a Bradford Protein Assay Kit (Sangon Biotech, Shanghai, China) based on the Coomassie brilliant blue method. Briefly, 0.1 g fresh powder were suspended in 5 mL phosphate buffer (pH 7.2) and centrifuged (8000× *g* for 10 min at 4 °C). The diluted supernatant was mixed with Coomassie brilliant blue R-250, after which the absorbance was measured at 595 nm. The total soluble protein content was recorded as mg/g FW.

The total amino acid content was measured with a commercial assay kit (Comin Biotechnology) based on a previously described method [41]. Briefly, 0.1 g fresh powder were dissolved in 1 mL glacial acetic acid, mixed thoroughly, and boiled for 15 min. After a centrifugation (8000× *g* for 5 min at 4 °C), the supernatant was added to a reaction solution consisting of 100 μL sodium acetate-glacial acetic acid, 100 μL ninhydrin, and 10 μL ascorbic acid. After boiling for 15 min, the absorbance of the reaction solution was measured at 570 nm, and the total amino acid content was recorded as mg/g FW.

### 2.6. Measurement of Organic Acids and Amino Acids

To analyze the organic acid and amino acid contents, 0.4 g fresh powder were suspended in 2 mL sterile water and boiled at 100 °C for 90 min. After a centrifugation, the supernatant was filtered (0.45 µm pore size). The organic acid and amino acid contents of the filtrate were analyzed by high-performance liquid chromatography with the 2695 Separations Module (Waters, Milford, MA, USA) as previously described [37,42]. All assays were completed with three biological replicates per treatment.

Organic acids: The citrate, succinate, and malate contents of the prepared extract were measured at 214 nm with the Waters 2487 UV detector, a Thermo ODS HYPERSIL (4.6 mm × 200 mm) chromatographic column, and 3% CH_3_OH + 97% H_2_O as the mobile phase (flow rate, 0.8 mL/min). The organic acid contents were recorded as mg/g FW.

Amino acids: The prepared extract was analyzed with a pre-column derivatized with the AccQ Fluor reagent. The mobile phase consisted of 140 mM NaAc solution (containing 17 mM triethylamine, pH 4.95) and a 60% aqueous acetonitrile solution. A Waters 2475 fluorescence detector and an AccQ Tag amino acid analysis column (3.9 mm × 150 mm) were used. The separate amino acids were detected at excitation and emission wavelengths of 250 and 395 nm, respectively. The amino acid contents were recorded as μg/g FW.

GABA: The prepared extract was mixed with acetonitrile containing 1% 2,4-dinitrofluorobenzene, after which an equal volume of NaHCO_3_ (pH 9) buffer was added, and the solution was incubated for 1 h at 60 °C. The solution contents were separated on a C_18_ SunFire column (4.6 mm × 250 mm), and the eluted products were detected with the Waters 2487 UV detector. The column temperature was maintained at 35 °C, and the mobile phase comprised phosphate buffer (pH 7), water, and acetonitrile, with a flow rate of 0.1 mL/min. The separated GABA was detected at 360 nm. The GABA content was recorded as μg/g FW.

### 2.7. Statistical Analysis

Data were compared and analyzed with ANOVA (analysis of variance), and multiple comparisons were made with SPSS 16.0 (SPSS, Chicago, IL, USA). Differences were scored as significant at the *p* < 0.05 or *p* < 0.01 levels. A principal component analysis (PCA) was performed with the command prcomp() in R (http://www.r-project.org/), as previously described [43,44].

## 3. Results

### 3.1. Effects of GABA and VGB on Seed Germination Characteristics

**Figure 1:**
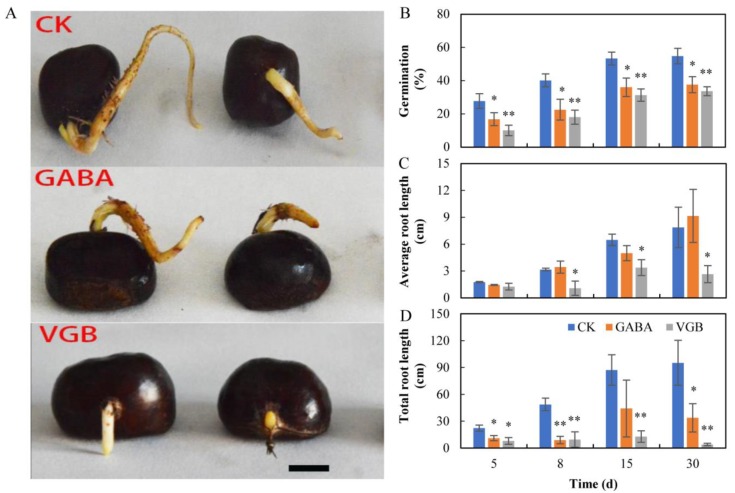
(**A**) Effects of exogenous GABA and VGB on (**B**) chestnut seed germination and (**C**,**D**) primary root growth. * and ** represent significant differences between the treatments and the control (CK) at *p* < 0.05 and *p* < 0.01, respectively.

The CK, 10 mM GABA, and 100 μM VGB solutions did not induce seed germination on Day 2. The germination rate of the CK seeds increased after five days, whereas the 10 mM GABA and 100 µM VGB treatments inhibited chestnut seed germination and primary root growth at five days after sowing (Figure 1A). The germination rates of the CK seeds gradually increased by over 27.8–54.8% between Days 5 and 30 (Figure 1B), whereas the germination rates gradually decreased by over 11.0–17.2% and 17.7–21.1% following the GABA and VGB treatments, respectively (Figure 1B). The VGB treatment decreased the average root length per seed between Days 8 and 30 (Figure 1C), whereas the total root length was generally decreased by both GABA and VGB between Days 5 and 30 (*p* < 0.05). These results indicated that both treatments had significant inhibitory effects on chestnut seed germination and early root growth.

### 3.2. Changes to Endogenous GABA and H_2_O_2_ Contents

**Figure 2:**
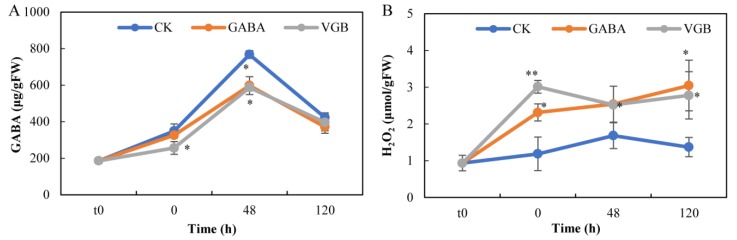
Effects of exogenous GABA and VGB on endogenous (**A**) GABA and (**B**) H_2_O_2_ contents during chestnut seed germination. * and ** represent significant differences between the treatments and control (CK) at *p* < 0.05 and *p* < 0.01, respectively. t0: time-point when the seed imbibition was initiated.

In the untreated seeds, the endogenous GABA concentration was initially 185.5 μg/g FW, but then increased to 767.3 μg/g FW at 48 h, after which it significantly decreased to 422.6 μg/g FW at 120 h (Figure 2A). However, the endogenous GABA concentrations decreased only following the VGB treatment after the 15 h imbibition (0 h). Additionally, the GABA and VGB treatments significantly decreased the endogenous GABA concentrations by over 22.1–23.5% at 48 h. Regarding the H_2_O_2_ contents, the CK level slightly increased. Surprisingly, the H_2_O_2_ contents increased considerably after the GABA and VGB treatments. After the 15 h imbibition, the H_2_O_2_ contents increased sharply by over 94.0% and 163.0% in response to GABA and VGB treatments, respectively, at 0 h, and remained high until 120 h (Figure 2B).

### 3.3. Effects of GABA and VGB on Carbon Metabolism

**Figure 3:**
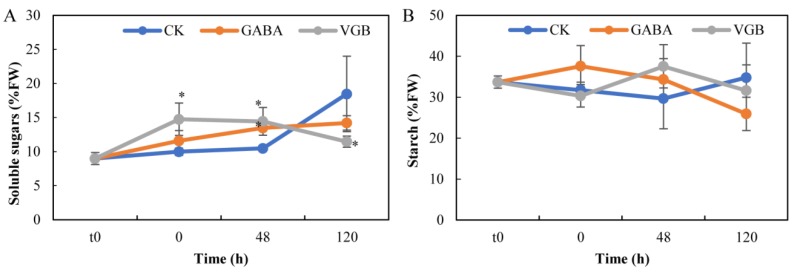
Effects of exogenous GABA and VGB on (**A**) the soluble sugar and (**B**) starch contents during chestnut seed germination. * represents a significant difference between the treatments and control (CK) at *p* < 0.05. t0: time-point when the seed imbibition was initiated.

**Figure 4:**
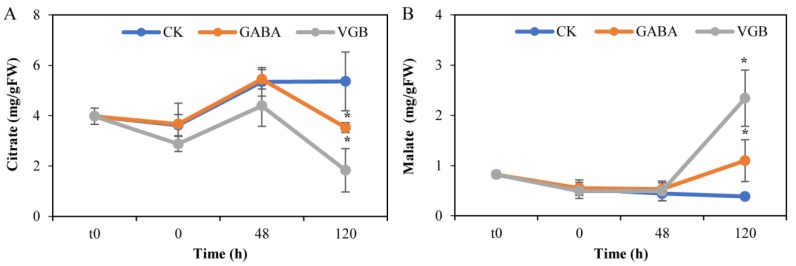
Effects of exogenous GABA and VGB on (**A**) the citrate and (**B**) malate contents during chestnut seed germination. * represents a significant difference between the treatments and control (CK) at *p* < 0.05. t0: time-point when the seed imbibition was initiated.

Compared with the CK level, the soluble sugar contents significantly increased in response to exogenous GABA only at 48 h, whereas it significantly increased at 0 and 48 h, but decreased at 120 h following the VGB treatment (Figure 3A). However, the GABA and VGB treatments did not significantly affect the starch contents (Figure 3B). Additionally, compared with the corresponding CK levels, both treatments decreased and increased the abundance of the tricarboxylic acid (TCA) cycle intermediates citrate and malate, respectively, but only at 120 h; there were no significant differences with the CK levels at all other examined time-points (Figure 4). Specifically, the citrate content decreased by over 34.2% and 65.8% (Figure 4A), and the malate content increased by over 1.8- and 5.1-fold (Figure 4B), in response to the GABA and VGB treatments, respectively. These results indicated that both treatments mainly affected the TCA cycle activity after germination.

### 3.4. Effects of GABA and VGB on Nitrogen Metabolism

**Figure 5:**
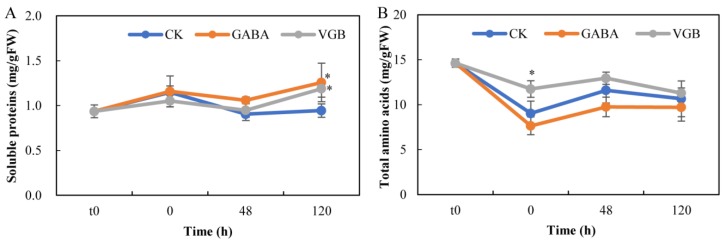
Effects of exogenous GABA and VGB on (**A**) soluble protein and (**B**) total amino acid contents during chestnut seed germination. * represents a significant difference between the treatments and control (CK) at *p* < 0.05. t0: time-point when the seed imbibition was initiated.

**Figure 6:**
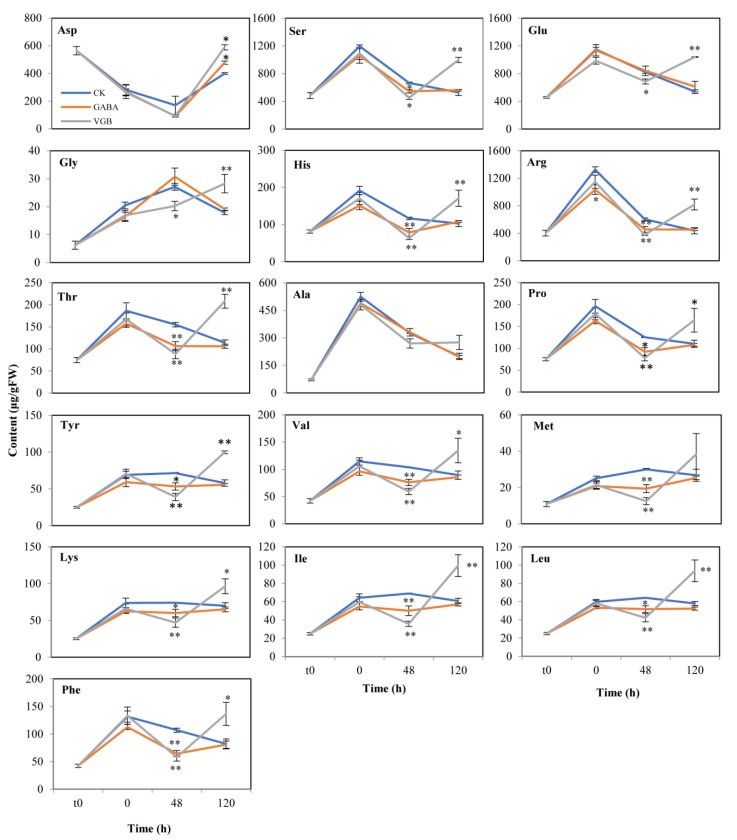
Effects of exogenous GABA and VGB on free amino acid contents during chestnut seed germination. * and ** represent significant differences between the treatments and control (CK) at *p* < 0.05 and *p* < 0.01, respectively. t0: time-point when the seed imbibition was initiated.

Relative to the CK levels, the soluble protein contents increased in response to exogenous GABA and VGB at 120 h (Figure 5A). In contrast, only VGB increased the total amino acid content (0 h) (Figure 5B). The abundance of the following 12 free amino acids decreased after the GABA and VGB treatments at the 48 h time-point: Ser, His, Arg, Thr, Pro, Tyr, Val, Met, Lys, Ile, Phe, and Leu. At 120 h, only the Asp content was increased by both treatments, whereas the abundance of 13 free amino acids increased only in response to VGB (i.e., Ser, Glu, Gly, His, Arg, Thr, Pro, Tyr, Val, Lys, Ile, Phe, and Leu) (Figure 6). These results implied that the GABA and VGB treatments negatively regulated amino acid metabolism before germination, but after germination, the free amino acid contents were increased mainly by VGB.

### 3.5. Analysis of the Physiological Response to Germination Following the GABA and VGB Treatments

**Figure 7:**
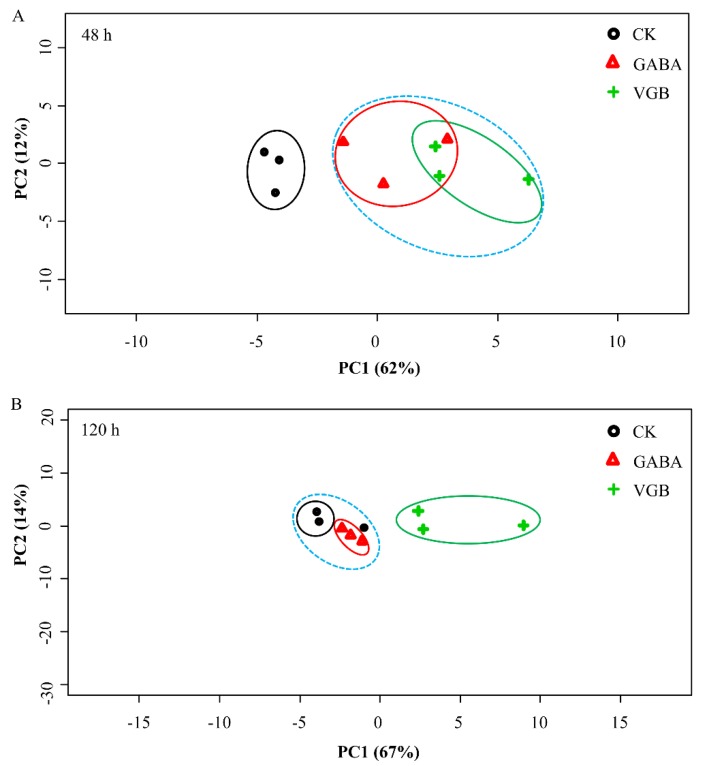
Principal component analysis of the effects of exogenous GABA and VGB on physiological parameters at 48 (**A**) and 120 h (**B**).

We completed a PCA to explore the effects of GABA and VGB before germination (48 h) (Figure 7A) and after germination (120 h) (Figure 7B). Specifically, the physiological traits of the chestnut seeds were evaluated (Table S1). Our data revealed that PC1 and PC2 accounted for 62% and 12% of the physiological variation before germination (48 h), respectively (Figure 7A). The effects of GABA and VGB were clearly separated from the effects of CK by PC1 at 48 h. Additionally, Ser, Pro, Tyr, Val, Met, and Ile were key contributors to PC1, whereas soluble sugars and total amino acids were important factors for PC2 (Table S1). After germination (120 h), PC1 and PC2 accounted for 67% and 14% of the physiological variation, respectively (Figure 7B). The effects of the VGB treatment were clearly separated from the effects of CK; however, the effects of the GABA treatment and CK were uncovered by PC1. These findings indicated that exogenous GABA and VGB were important for inhibiting chestnut seed germination.

## 4. Discussion

Chestnut seeds are widely considered to be healthy for humans [45]. Earlier reports regarding the substantial accumulation of GABA in chestnut seeds [7,36] provided evidence of the health benefits of chestnuts, similar to other GABA-enriched functional foods [9,10]. Additionally, seed germination is closely associated with the shelf-life of chestnuts and with the cultivation of rootstock seedlings [30]. Therefore, it is worth considering the high GABA level regarding its effects on the storage and germination of chestnut seeds.

Recent genetic and physiological studies have implied that GABA is involved in responses to abiotic stresses [46,47], as well as developmental processes, including pollen tube growth [48], primary/adventitious root growth [37,49], and seed germination [20,21]. The recent identification of a GABA receptor, aluminum-activated malate transporter (ALMT) [50], indicates that GABA is a signaling molecule and not just a metabolite [51]. Consequently, GABA functions should be more comprehensively characterized.

Previous studies revealed that endogenous GABA concentrations increase during germination [16,17,52]. In contrast, in the current study, we observed that germination was negatively related to endogenous GABA concentrations at the 48 and 120 h time-points following the GABA and VGB treatments (Figure 1A and Figure 2A). Our results were similar to those of an earlier investigation on high lysine maize seeds [13]. We also detected a more than four-fold increase in GABA concentrations before germination (Figure 2A), which might result from the decrease in Glu content at 48 h (Figure 6) because Glu acts as the direct precursor of GABA production [8,51]. This further indicated that chestnut seeds may be useful as a GABA-enriched functional food by short-term germination induction. However, the decrease in GABA after 48 h may be ascribed to the requirement of much more Glu for protein synthesis or rapid degradation of GABA back to the TCA cycle during primary root growth [8,51]. However, the application of 10 mM GABA inhibited chestnut seed germination and early primary root growth (Figure 1), which was inconsistent with the results of earlier investigations on barley [20] and *H. ammodendron* [21] seeds. Moreover, blocking GABA degradation with 100 μM VGB also had an inhibitory effect (Figure 1). We recently confirmed VGB to be detrimental to adventitious root growth [37]. Thus, the endogenous GABA may play a specific role in the germination of chestnut seeds and may be useful for improving chestnut seed storage during winter.

Generally, exogenous GABA promotes germination [20,21] and enhances GABA absorption in orthodox seeds [20]. Unexpectedly, our analysis of recalcitrant chestnut seeds uncovered a transient decrease in endogenous GABA concentrations at 48 h following the GABA and VGB treatments (Figure 2A), which was inconsistent with the data generated during our recent investigation of GABA- and VGB-treated poplar stem fragments [37]. Previous studies proved that embryo axes and cotyledons exhibited contrasting responses to desiccation in *Castanea sativa* seeds [27], and the embryonic axes of dormant seeds were maintained in a state of metabolic readiness under optimal conditions [29]. Accordingly, it is possible that the results of the current study were due to the embryos being highly sensitive to the imbibition of exogenous GABA and VGB, resulting in significant increases in endogenous GABA concentrations, relative to the levels of cotyledon with high moisture. However, the whole seed kernels used for measurements may have obscured the final increased GABA concentrations in the embryos. Additionally, GABA provides the carbon skeletons in the TCA cycle [8], which contributes to seed germination [20]. In this study, the GABA and VGB treatments altered the metabolism of soluble sugars, organic acids, and amino acids.

Carbon and nitrogen metabolites, including those mentioned above, are significantly associated with germination and seedling establishment [13], during which GABA critically affects carbon and nitrogen metabolism [8]. During seed germination, as the hormone GA’s role [53], exogenous GABA may promote starch hydrolysis to produce soluble sugars by stimulating α-amylase activity [20], but in the current study, there were no significant changes in starch contents following the GABA and VGB treatments (Figure 3B). This may have resulted in the decrease in soluble sugar contents at 120 h (Figure 3A), with the resulting lack of sufficient energy leading to the inhibition of early primary root growth (Figure 1). However, we speculated that the increase in soluble sugar levels at 48 h may have resulted from the lipid breakdown occurring in germinating seeds [53]. We observed that both treatments induced the considerable accumulation of H_2_O_2_ (Figure 2B), which can be mainly produced by fatty acid β-oxidation during germination [53], contributing to the inhibition of chestnut seed germination.

The TCA cycle activity is closely associated with seed germination [13], wherein succinate is also the final product of GABA degradation [8]. However, the succinate contents could not be detected in this study. Previous research demonstrated that increasing lysine levels in *Arabidopsis thaliana* seeds resulted in delayed germination, which was accompanied by a significant decrease in the levels of TCA cycle metabolites, such as citrate, malate, and succinate [13]. Hence, the observed decrease in citrate contents at 120 h (Figure 4A) may adversely affect germination and early primary root growth in chestnut seeds because of the associated lack of sufficient energy. Unlike the study by Angelovici et al. [13], we detected an increase in malate contents induced by GABA and VGB treatments (Figure 4B). This increase may block the germination and early primary root establishment of chestnut seeds, which is supported by a recent report [54], which proved that rapidly germinating seeds have low malate levels. Because malate is a key intermediate of the TCA cycle, we speculated that the accumulation of malate may affect the efficient mobilization of storage compounds to supply energy for germination and early seedling development. However, the results of our recent study implied that malate interacting with GABA can delay poplar AR formation [37], possibly because GABA can negatively modulate ALMT via malate [50,55,56]. Thus, we considered that changes to the metabolic status of malate and GABA led to physiological responses, such as the delayed seed germination and inhibited early primary root growth observed in this study, through modulated ALMT activities.

In many plant species, most amino acids accumulate during seed germination [12]. For example, the aspartic acid family of amino acids contributes to the onset of autotrophic growth-associated processes during germination [13]. An exogenous nitric oxide donor (*S*-nitroso-*N*-acetyl-d,l-penicillamine) can enhance the germination of Kabuli chickpea seeds, which coincides with an increase in amino acid levels [57]. Exogenous H_2_O_2_ also promotes the germination of eggplant seeds, accompanied by enhanced amino acid biosynthesis and protein expression [58]. Therefore, the observed decrease in most of the amino acid levels at the 48 h time-point following the GABA and VGB treatments might contribute to the inhibition of chestnut seed germination (Figure 6). At 120 h, however, only the VGB treatment induced a considerable increase in the accumulation of 10 amino acids (Figure 6), which were reportedly negatively associated with root growth [13,37,58,59,60]. Thus, VGB might have a specific role in inhibiting early primary root growth. However, the GABA treatment did not adversely affect the roots, which was consistent with the findings of a previous study involving *Brassica napus* seedlings [60], but it inhibited chestnut seed germination. Furthermore, soluble protein contents reportedly decreased during the seed germination of six grass species [61], but they increased significantly when the primary root growth was inhibited at 120 h after both treatments in the current study (Figure 5). Therefore, the two treatments appeared to negatively influence early primary root growth in chestnut seeds.

## 5. Conclusions

**Figure 8:**
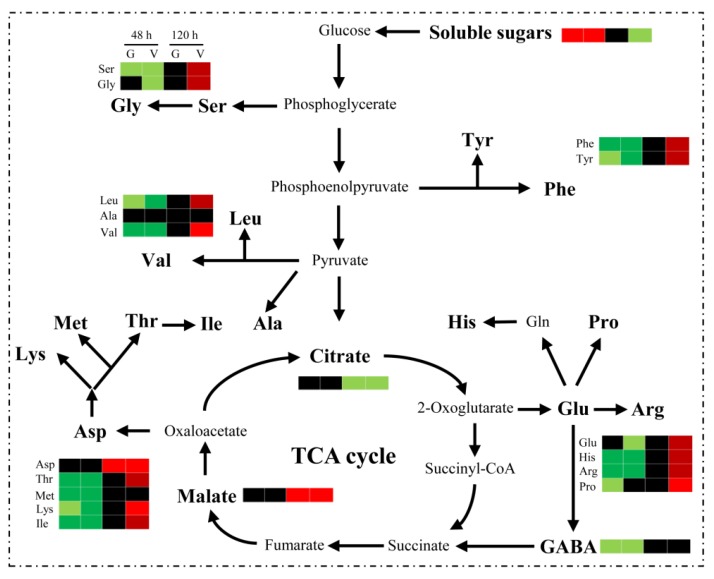
Model of the effects of exogenous GABA and VGB on primary carbon and nitrogen metabolism during chestnut seed germination at 48 and 120 h. Red: increase; green: decrease; black: no significant change; G: GABA vs. CK; V: VGB vs. CK.

The importance of GABA for human health and plant development has been confirmed. In this study, high GABA levels were detected in seeds before germination, implying GABA not only could influence chestnut seed germination, but also could act as a potential compound of functional chestnut food. This should be examined in greater detail in future studies. The application of exogenous GABA and VGB inhibited chestnut seed germination and early primary root growth, possibly by altering the balance between carbon and nitrogen metabolism, especially the free amino acid contents before germination (Figure 8). The data presented herein suggested that changes to the endogenous GABA levels in chestnut seeds might adversely affect germination. This insight may be relevant for improving the storage of chestnut and other recalcitrant seeds over winter.

## Supplementary Materials

The following are available online at https://www.mdpi.com/2223-7747/9/4/449/s1, Table S1: Data of PCA analysis of exogenous GABA and VGB on the physiological effects during chestnut seed germination.
